# Human Hepatocyte Growth Factor Promotes Functional Recovery in Primates after Spinal Cord Injury

**DOI:** 10.1371/journal.pone.0027706

**Published:** 2011-11-29

**Authors:** Kazuya Kitamura, Kanehiro Fujiyoshi, Jun-ichi Yamane, Fumika Toyota, Keigo Hikishima, Tatsuji Nomura, Hiroshi Funakoshi, Toshikazu Nakamura, Masashi Aoki, Yoshiaki Toyama, Hideyuki Okano, Masaya Nakamura

**Affiliations:** 1 Department of Orthopedic Surgery, Keio University School of Medicine, Shinjuku-ku, Tokyo, Japan; 2 Department of Physiology, Keio University School of Medicine, Shinjuku-ku, Tokyo, Japan; 3 Department of Orthopedic Surgery, Hiratsuka City Hospital, Hiratsuka, Kanagawa, Japan; 4 Central Institute for Experimental Animals, Miyamae-ku, Kawasaki, Kanagawa Japan; 5 Center for Advanced Research and Education, Asahikawa Medical University, Midorigaoka, Asahikawa, Japan; 6 Kringle Pharma Joint Research Division for Regenerative Drug Discovery, Center for Advanced Science and Innovation, Osaka University, Osaka, Japan; 7 Department of Neurology, Tohoku University School of Medicine, Sendai, Miyagi, Japan; University of South Florida, United States of America

## Abstract

Many therapeutic interventions for spinal cord injury (SCI) using neurotrophic factors have focused on reducing the area damaged by secondary, post-injury degeneration, to promote functional recovery. Hepatocyte growth factor (HGF), which is a potent mitogen for mature hepatocytes and a mediator of the inflammatory responses to tissue injury, was recently highlighted as a potent neurotrophic factor in the central nervous system. We previously reported that introducing exogenous HGF into the injured rodent spinal cord using a herpes simplex virus-1 vector significantly reduces the area of damaged tissue and promotes functional recovery. However, that study did not examine the therapeutic effects of administering HGF after injury, which is the most critical issue for clinical application. To translate this strategy to human treatment, we induced a contusive cervical SCI in the common marmoset, a primate, and then administered recombinant human HGF (rhHGF) intrathecally. Motor function was assessed using an original open field scoring system focusing on manual function, including reach-and-grasp performance and hand placement in walking. The intrathecal rhHGF preserved the corticospinal fibers and myelinated areas, thereby promoting functional recovery. *In vivo* magnetic resonance imaging showed significant preservation of the intact spinal cord parenchyma. rhHGF-treatment did not give rise to an abnormal outgrowth of calcitonin gene related peptide positive fibers compared to the control group, indicating that this treatment did not induce or exacerbate allodynia. This is the first study to report the efficacy of rhHGF for treating SCI in non-human primates. In addition, this is the first presentation of a novel scale for assessing neurological motor performance in non-human primates after contusive cervical SCI.

## Introduction

Spinal cord injury (SCI) is followed by secondary degeneration, which is characterized by progressive tissue necrosis. Many experimental interventions have focused on this posttraumatic inflammatory process, using neurotrophic factors to reduce the damaged area and to promote axonal regeneration through the lesion epicenter. Neurotrophins such as nerve growth factor (NGF) [1], [2], brain-derived neurotrophic factor (BDNF) [3], neurotrophin-3 (NT-3) [4], [5], and glial cell line-derived neurotrophic factor (GDNF) [6], [7] have been reported to enhance axonal growth in the injured spinal cord; some of these studies also showed that neurotrophins promoted behavioral recovery after SCI [3], [7]. Both neurotrophic support and angiogenesis are critical to the endogenous regenerative response to trauma after SCI [8], [9]. The initial damage to local blood vessels is decisive for the progression of destructive events during secondary degeneration [10], and strategic treatments to improve angiogenesis after SCI have shown a relationship between blood flow and functional recovery [11], [12].

Hepatocyte growth factor (HGF) was first identified as a potent mitogen for mature hepatocytes [13], [14] and a natural ligand for the c-Met proto-oncogene product [15]. Recent studies have revealed that HGF acts as a neurotrophic factor for a variety of neuron types [16], [17], [18], [19], [20], and that administering HGF enhances angiogenesis, improves microcirculation, inhibits destruction of the blood-brain barrier [21], and exerts a neuroprotective effect after cerebral ischemia [22], [23] and in the transgenic amyotrophic lateral sclerosis (ALS) rat model [24]. We previously reported that introducing exogenous HGF into the spinal cord significantly reduces the damaged area and promotes functional recovery in adult rats [25]. However, this strategy, which involved injecting a herpes simplex virus-1 vector into the spinal cord prior to SCI, can never be applied to clinical treatment. Furthermore, rodent SCI models are limited in their ability to ensure the efficacy and safety of treatments for humans. It is critical to examine potential treatments in non-human primates before proceeding to clinical trials.

In this study, a total of 400 µg of recombinant human HGF (rhHGF) was infused intrathecally for four weeks immediately after C5-level contusive SCI in adult common marmosets, as a preclinical trial. Contusive injury is considered the most relevant to human SCI when evaluating therapies and predicting their translation to human treatment. However, to assess hand dexterity recovery, we needed an open field rating scale for a primate cervical SCI model that would be comparable to the BBB scale in rodents [26]. Therefore, we established an original open field rating scale to evaluate hand neurological performance after contusive cervical SCI. We found that intrathecal rhHGF infusion significantly reduced the areas of damaged tissue in the spinal cord and promoted functional recovery, consistent with our previous study using rats. This is the first study to report the efficacy and safety of rhHGF for SCI, and to present a novel scale for assessing neurological motor performance after contusive SCI in non-human primates.

## Results

### Distribution of the spinal motoneurons regulating wrist and finger motion in common marmosets

To precisely evaluate motor functions in the marmoset SCI model, we focused on the wrist and finger extension movements, because these motions are the most impaired in incomplete cervical SCI [27].

**Figure 1:**
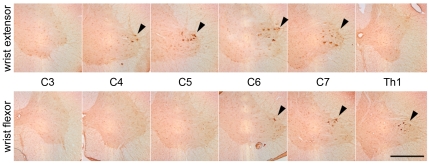
Retrograde labeling of motoneurons in the intact spinal cord by CTB injection. The motoneurons innervating the wrist and finger flexors and extensors were mainly distributed in lamina IX of the C4–C7 segments and the C6-Th1 segments, respectively. Almost all of these motoneurons were located at sites caudal to the lesion epicenter at the C5 level. Scale bar, 500 µm.

We first investigated the distribution of spinal motoneurons that innervate the hand muscles in marmoset forelimbs, by injecting cholera toxin B subunit (CTB) into the forearm flexor and extensor muscles. The wrist and finger extensor motoneurons were mainly located in lamina IX of the cervical (C)4-C7 segments, and wrist flexor motoneurons were in lamina IX of the C6-thoracic (Th)1 segments (Fig. 1). Based on these findings, we quantified the number of ChAT-positive motoneurons at these segments in marmosets treated with rhHGF or with sterile phosphate-buffered saline (PBS) after SCI. Twelve weeks after injury, the ChAT-positive motoneurons around the lesion epicenter had almost disappeared, consistent with our previous report [28], and we did not detect any significant differences in the number of surviving C2-Th1 ChAT-positive motoneurons between the two groups (data not shown).

### rhHGF significantly preserved the corticospinal tract pathway and LFB-positive myelinated areas

**Figure 2:**
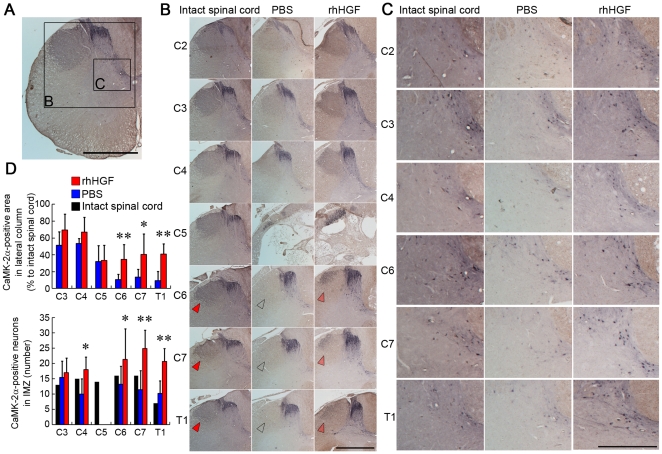
CaMK II α-immunoreactivity in the marmoset spinal cord. CaMK II α-immunoreactivity (IR) in the lateral column of the white matter and in the intermediate zone (IMZ) of the gray matter in an intact spinal cord (A) and injured spinal cord 12 weeks after SCI (B, C). (A) In the intact spinal cord, CaMK II α-IR was detected in the lateral column, dorsal horn and intermediate zone of the gray matter. No CaMK II α-IR was detected in the ventral horn of the gray matter. Scale bar, 1 mm. (B) CaMK II α-IR in the lateral column, suggesting the corticospinal tract (CST) pathway, was significantly preserved even at sites caudal to the lesion epicenter (arrowheads) in the rhHGF group compared with the PBS group. Scale bar, 1 mm. (C) CaMK II α-IR was detected in cell bodies at the IMZ (laminae VI–VII), suggested to be segmental interneurons and propriospinal neurons. There were significantly more IMZ CaMK II α-positive neurons at the C4 and C6-Th1 segments in the rhHGF group than in the PBS group. IMZ CaMK2-α-positive neurons were absent from the C5 segment in both groups. Scale bar, 500 µm. (D) Quantitative analyses of the CaMK II α-IR showed significant differences between the two groups. *P<0.05, **P<0.01. (n = 6 for the rhHGF group; n = 5 for the PBS group; n = 1 for the intact spinal cord).

We next investigated the pattern of the corticospinal tract (CST) pathway and its terminations in common marmosets. Axial sections of intact and injured spinal cords were immunostained with an anti-calmodulin-dependent kinase IIα (CaMK IIα) antibody [29]. We previously found that CaMK IIα-immunoreactivity (IR) labels the CST in the marmoset spinal cord [28], [30], [31], although its specificity remains to be demonstrated. In the present study, we made the following observations. First, in the intact spinal cord, CaMK IIα-IR was detected in the lateral column of white matter, the dorsal horn, and the intermediate zone (IMZ) of the gray matter (Fig. 2A), suggesting that the CST in the common marmoset is located in lateral columns, unlike in rodents, in which the CST projects mainly to dorsal horn neurons and premotor spinal circuits [32]. Second, we did not detect CaMK IIα-IR in either the ventral white matter column or the ventral gray matter horn, suggesting that, as in rodents, the proportion of the ventral CST extending into the white matter is small, and that the CST does not project directly to cervical motoneurons in the ventral horns.

Whereas CaMK II α-IR in the lateral column was obviously reduced in segments caudal to the lesion site in the PBS group compared with the intact spinal cord control, the CaMK II α-IR was well maintained, even caudal to the lesion site, in the rhHGF group (Fig. 2B arrowheads). Stereological quantification of the CaMK II α-positive area in the lateral column showed significant differences between the two groups at the segments caudal to the lesion site (Fig. 2D). CaMK II α-IR was detected in the IMZ (laminae VI–VII) cell bodies, which could include segmental interneurons (sINs) and propriospinal neurons (PNs). CaMK II α-positive neurons in the IMZ at C5 had disappeared at 12 weeks after SCI in both groups (Fig. 2B and C). The rhHGF group had obviously greater numbers of these neurons around the lesion epicenter, as compared with the PBS group (Fig. 2C). Quantitative analysis of these neurons showed significant differences between the two groups at the C4 and C6-Th1 segments (Fig. 2D). Note that in some segments, the number of neurons was greater in the rhHGF and PBS groups than in the intact spinal cord (Fig. 2D).

**Figure 3:**
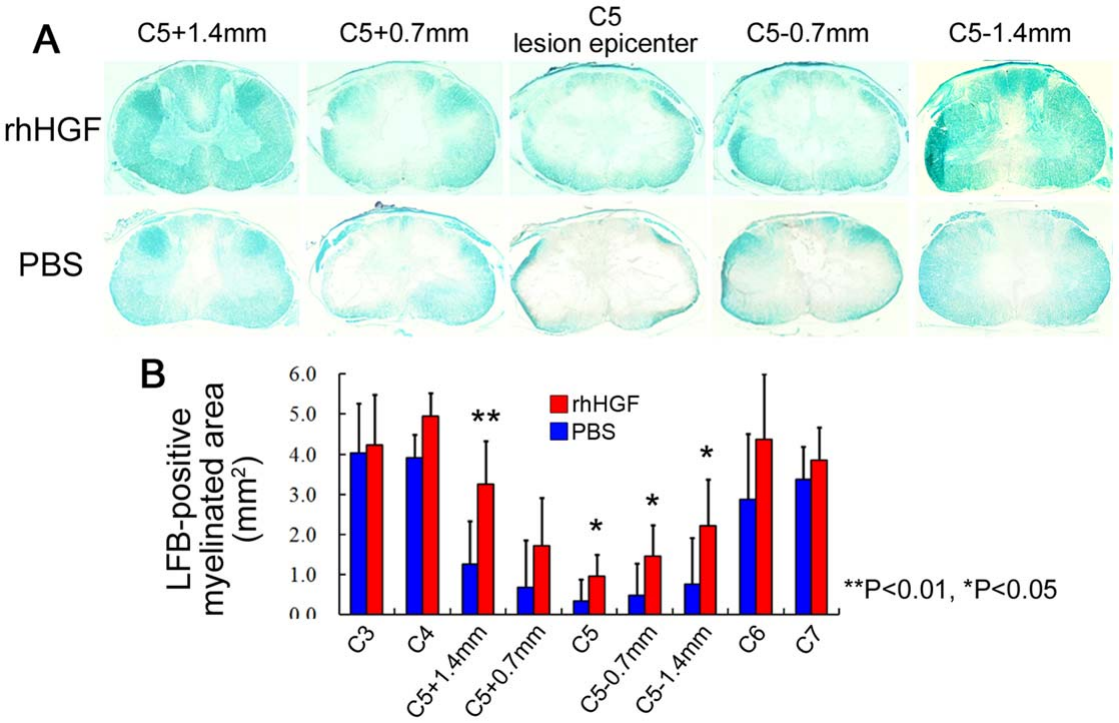
Significant reduction in the size of damaged parenchyma in the rhHGF group. Quantification of the LFB-positive myelinated area 12 weeks after SCI (A) showed a significant difference between the two groups around the lesion epicenter (B). Scale bar, 2 mm. *P<0.05, **P<0.01. (n = 6 for the rhHGF group; n = 5 for the PBS group).

To examine rhHGF's tissue-sparing effects, we stained axial sections with Luxol fast blue (LFB) 12 weeks after SCI. There was a significant rim of spared myelinated white matter, even at the lesion epicenter, in the rhHGF group (Fig. 3A). Stereological quantitative analysis of the LFB-positive myelinated areas revealed significant differences around the lesion epicenter between the two groups (Fig. 3B). Significant differences were observed within one vertebral segment from the lesion site at the C5 level.

### *In vivo* MRI and DTT findings reflected the histological severity of the spinal cord injury

**Figure 4:**
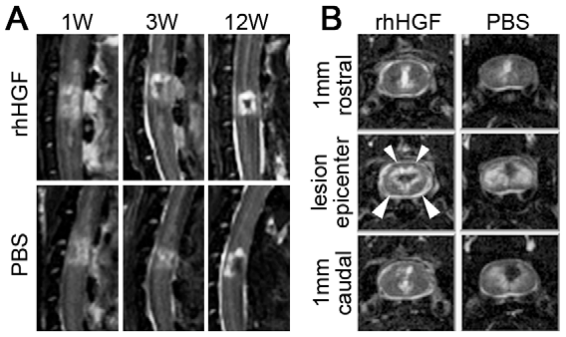
Representative MR images around the lesion epicenter after SCI, and correlation with functional recovery. (A) S Sagittal T2-weighted images around the lesion epicenter 1, 3, and 12 weeks after SCI. A marked reduction in the T2 low-signal-intensity area surrounded by a T2 high-signal-intensity area, suggesting cavity and glial scar formations, was detected in the rhHGF-treated animal compared with the vehicle control animal 12 weeks after SCI, even though there were no obvious differences between them up to 3 weeks after SCI. (B) Axial MR images around the lesion epicenter 12 weeks after SCI. The damaged T2 high-signal-intensity areas (arrowheads) were obviously reduced in the rhHGF-treated animal.

*In vivo* MRI and diffusion tensor tractography (DTT) [30] were conducted at multiple time points (1, 3, and 12 weeks after SCI) in four animals, two from each group that underwent SCI, to investigate *in vivo* images that might reflect the pathological changes after SCI. One animal from each group was subjected to MRI and DTT analysis on the same day, and the recovery of motor function was assessed over time using our open field rating scale. Sagittal T2-weighted images (T2WIs) from each individual showed a diffusely expanded high-intensity area around the lesion epicenter 1 week after SCI, which was gradually compacted thereafter with the low-intensity area inside (Fig. 4A). At 12 weeks after SCI, the area with abnormal intensity (low-intensity area surrounded by high-intensity area), suggesting a region of damaged parenchyma, was markedly reduced in the rhHGF-treated animal compared with the vehicle control animal (Fig. 4A). The axial T2WIs also showed marked reduction in the area with abnormal-intensity area. Note that the ventral and dorsal parts of the white matter in the rhHGF-treated animal showed normal intensity (Fig. 4B arrowheads) even at the lesion epicenter, whereas the abnormal T2-high intensity area in both the gray and white matter was greatly expanded in the vehicle control animal. The rhHGF-treated animal showed remarkable motor function recovery beginning 3 weeks after SCI; until that point there were no obvious differences in the MRIs between the two animals (Fig. 4A and B), corresponding with the time of functional recovery as assessed by our original open field rating scale. The open field scores for the upper limbs of the rhHGF-treated animal and the vehicle control animal were, respectively, 3 and 6 at 3 weeks, and 14.5 and 6.5 at 12 weeks after SCI.

**Figure 5:**
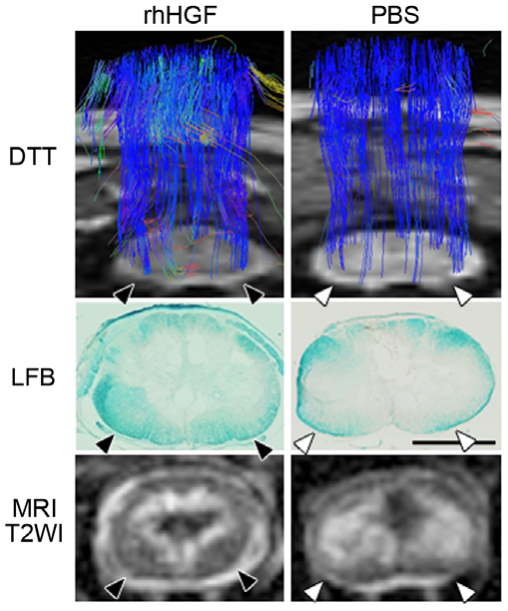
Diffusion tensor tractography (DTT) showing spinal fibers around the lesion epicenter, correlating with the histology and MR images. The region of interest (ROI) was placed in the upper cervical spinal cord, and full-width DTT of the spinal cord was traced in the caudal direction. Spinal fibers revealed by DTT in the ventral white matter (arrowheads) 12 weeks after SCI were correlated with the spared rim of LFB-positive myelinated area and the intact signal-intensity area in the axial T2-weighted images. Scale bar, 2 mm.

*In vivo* diffusion tensor analyses were performed at the same time points, and no obvious differences were observed between the two groups until 3 weeks after SCI (data not shown). At 12 weeks after SCI, DTT showed a larger number of spinal tract fibers around the lesion epicenter in the rhHGF group than in the PBS group, especially in the ventral white matter, which may have been a precise reflection of the spared rim of myelinated white matter detected by LFB staining and axial T2WIs (Fig. 5).

### rhHGF did not give rise to abnormal CGRP-positive fiber outgrowth

**Figure 6:**
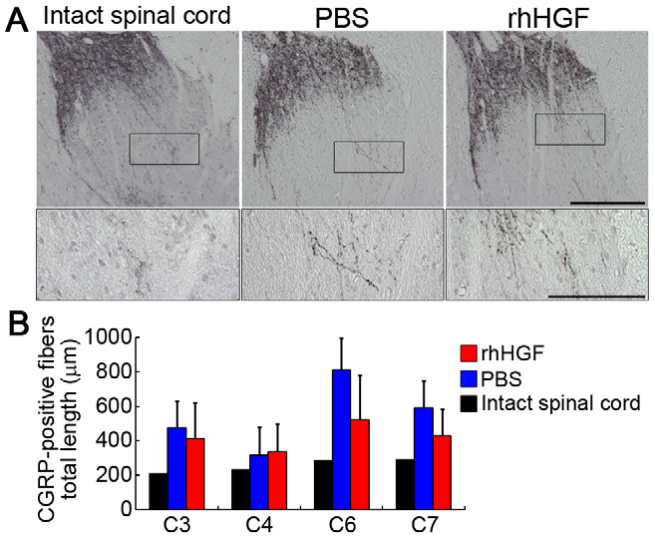
Quantitative analysis of CGRP-positive fibers at the dorsal horns. (A) Abnormal sprouting into lamina III at the dorsal horns was detected in both groups 12 weeks after SCI. Scale bar, 200 µm upper, 100 µm lower. (B) Quantitative analysis of the total length of these abnormal fibers showed no significant differences between the two groups in any segment examined (n = 6 for the rhHGF group; n = 5 for the PBS group; n = 1 for intact spinal cord).

Although allodynia-like withdrawal responses to mechanical stimulation of the four extremities were not observed after SCI in either group, we quantified the calcitonin gene-related peptide (CGRP)-immunoreactive sensory axons in the Rexed lamina III 12 weeks after SCI to determine whether rhHGF treatment induced histological changes that could cause allodynia. Dense CGRP-positive innervation of lamina I and the outer part of lamina II was observed before and after SCI, and abnormal innervations of lamina III were detected after SCI in both groups (Fig. 6A). Stereological quantification of the total length of CGRP-positive fibers in lamina III revealed increased CGRP-positive fiber length in both groups compared to the intact spinal cord, but did not show significant differences between the two groups rostrocaudal to the lesion epicenter at any site examined (Fig. 6B), suggesting that rhHGF treatment did not give rise to the abnormal sprouting of CGRP-positive nerve fibers.

### Motor function recovery over time, original scale development, and category definitions

**Table 1:** Original open field rating scale.

UPPER LIMBS maximum 20 points							
1. WEIGHT BEARING							
**weight bearing in stance**			anterior chest on the floor, no weight support of upperlimbs				+0
			anterior chest lifted up from the floor with weight support of upperlimbs				+1
**weight bearing in walking**			anterior chest on the floor, no weight support of upperlimbs				+0
			anterior chest lifted up from the floor with weight support of upperlimbs,				
			with forearm dragged				+1
			with hand dragged				+1
			stepping with hand clearance from the floor				+1
			coordinated steps with lowerlimbs				+1
**hand position in walking**			below shoulder				+0
			between shoulder and head				+1
			above head				+1
**hand placement in walking**			dorsal placement (dropped wrist)				+0
			ulnar placement, not pronated				+1
			pronated, but no palmar placement				+1
			pronated with palmar placement				+1
2. REACH AND GRASP PERFORMANCE							
			elevation below head-height				+0
			cannot grasp a pen			+0	
			grasp a pen			+1	
			elevation up to head-height			+1	
			cannot grasp a pen at head-height			+0	
			grasp a pen perpendicular to the body plane				+1
			grasp a pen parellel to the body plane				+1
			elevation up to 3cm above head-height			+1	
			cannot grasp a pen at 3 cm above head-height				+0
			grasp a pen perpendicular to the body plane				+1
			grasp a pen parellel to the body plane				+1
3. SOMATOSENSORY FUNCTION							
**somatosensory**			drop upper limbs through gaps in the cage bars up to				
			humerus			+0	
			elbow			+1	
			hand only			+1	
			never drop			+1	

TRUNK STABILITY maximum 3 points				
			cannot roll-over from supine to prone position	+0
			roll-over from supine to prone position	+1
			keep sitting position	+1
			keep standing position	+1

LOWER LIMBS maximum 7 points			
**ROM & weight bearing**		slight movement of lowerlimbs	+0
		extensive movement of lowerlimbs	+1
		move forward with weight bearing of lowerlimbs	+1
		jump	+1
		jump successively	+1
**somatosensory**		drop lower limbs through gaps in the cage bars up to	
		thigh	+0
		knee	+1
		foot only	+1
		never drop	+1

**Definitions and abbreviations.**

<***UPPERLIMBS***>** dorsal placement**: wrist joint is dropped and dorsal side of the hand is placed on the floor; **ulnar placement**: pronation of forearm is insufficient and little finger of the hand (ulnar side of the hand) is placed on the floor; **pronated, but no palmar placement**: forearm is pronated, wrist joint is extended, but no palmar placement due to limitted fingrs extension; **pronated with palmar placement**: palmar placement with forearm pronated and wrist and fingers extended <***TRUNK STABILITY***>** keep sitting or standing position**: keep sitting or standing position more than 5 seconds in the cage <***LOWER LIMBS***>** ROM**: range of motion; **slight movement of lowerlimbs**: partial joint movement through less than half of the range of the three joints motion; **extensive movement of lowerlimbs:** movement through more than half of the range of the three joints motion.

All animals showed severe quadriplegia 1 day after injury; they lay on the floor in a prone position, with little limb movement, and could not roll over or move forward by themselves. After C5-level SCI, irrespective of therapeutic intervention, the animals showed progressive recovery of motor function as assessed in three main categories: upper limbs, lower limbs, and trunk stability (Table 1). This study's injury model, cervical SCI, requires a detailed analysis of upper limb function to precisely evaluate motor function recovery. Thus, the upper limb category was divided into three subdivisions: weight bearing, reach and grip performance, and somatosensory function (Table 1). Because there were various combinations of improvement in multiple subdivisions and attributes, we adopted a point-addition scoring system in this original open field rating scale, unlike the system used in the BBB scale [26].

#### Weight bearing

**Figure 7:**
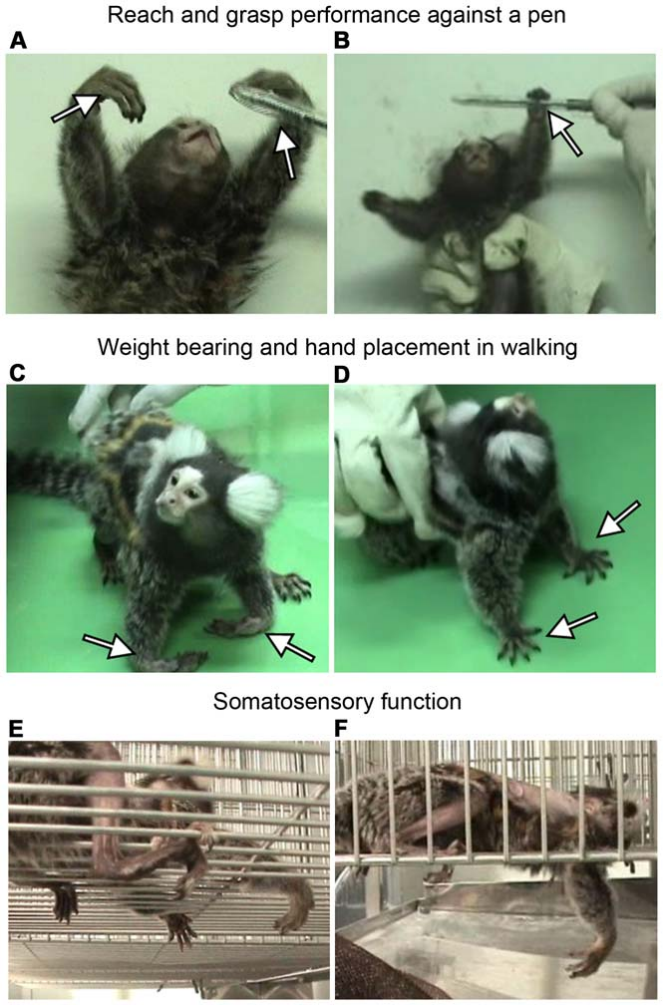
Representative images of marmosets after SCI. (A and B) An animal in the PBS group showed good elevation of upper limbs up to the head level 8 weeks after SCI. However, this animal could not grasp a pen parallel to the body plane because of insufficient forearm pronation and wrist and finger extension (A, arrowheads). An animal in the rhHGF group could reach for a pen at 3 cm above head height, and could grasp a pen even when parallel to the body plane. Note that the forearm was well pronated, with the wrist joint fully extended (B arrowhead). (C and D) The same animal as in (A) walked with the anterior chest lifted up from the floor by bearing its weight on the upper limbs. However, the wrist joints dropped completely, and the dorsal surfaces of the hands were placed and dragged on the floor (C arrowheads). On the other hand, the same animal as in (B) showed good palmar placement of the hands, with the wrist joints completely extended (D arrowheads), and walked with its hands clear of the floor. (E and F) One week after SCI, an animal in the PBS group dropped its limbs through gaps in the cage floor as far as the humerus or thigh, and could not raise the dropped limb quickly (F). The animal gradually recovered, and at 6 weeks after SCI, rarely dropped its limbs to the humerus or thigh level, but still often dropped them as far as the elbow or knee (E).

The early phase of recovery was characterized initially by the animals' movement and placing of weight on the lower limbs. As the animals recovered, they showed slight to extensive movements of the three lower limb joints, and began to lift their upper body from the floor by bearing weight on the upper limbs in stance only. They also began to move forward by placing weight on the lower limbs, dragging the anterior chest on the floor without other observable movements, and placing weight on the upper limbs with the hands below shoulder level. The next phase of recovery was characterized by lifting the upper body by placing weight on the upper limbs, even when moving forward. When the animals progressed to raising the upper body from the floor in walking, they began to move their upper limbs to the rhythm of the walk, with their hands above shoulder level. However, more typically, the forearms were dragged on the floor, with the wrist joints dropped and the forearms not pronated. They were eventually able to bear weight on the upper limbs while walking, dragging only the hands, and finally recovered to walking without dragging the hands, in the most-recovered cases in the rhHGF group. The hand position also gradually recovered from being between the shoulder and head level to above the head. The most remarkable category, hand placement in walking, was assessed when the hands were above shoulder level, and was characterized by wrist extension, forearm pronation, and finger extension. Initially, the wrist joints were completely dropped, and the forearm was not pronated; thus, the animals supported their weight on the dorsal side of the hands (Fig. 7C) or on the ulnar side of the upper limbs. They eventually were able to extend the wrist joints, pronate the forearms, extend the fingers, and reestablish palmar contact with weight support (Fig. 7D); however no animals in the PBS group attained this level of recovery. The best performance was judged by the animal's ability to maintain a particular pattern throughout the testing session, not by its ability to exhibit a behavior once.

#### Reach-and-grasp performance

The assessment of reach-and-grasp performance highlighted finger flexion and extension, wrist extension, forearm pronation, and shoulder flexion. For several days after SCI, the animals could not raise their upper limbs, and could not actively flex their fingers even when a pen was placed in their hands. The animals eventually began to grasp a pen passively placed in their hands, and to lift their upper limbs to reach and grasp a pen that was suddenly presented in front of their eyes. They typically recovered the ability to elevate their hands up to head height, and to reach and grasp a pen perpendicular to the body plane, with incomplete extension of the fingers and wrist joint. Many animals did not recover sufficiently to grasp a pen parallel to the body plane, even at head height (Fig. 7A), because it required full forearm pronation. They were also unable to elevate their hands to 3 cm above head height or to grasp pens in both directions, as this required active full shoulder joint flexion and full finger and wrist extension (Fig. 7B). In this assessment, the best performance was determined to be those attributes that were closest to normal behavior, even if they occurred only once.

#### Somatosensory function

Whereas intact animals never dropped their limbs through the gaps in the cage bars, animals after SCI often dropped the limbs through the floor gaps. We evaluated the distance the limb was dropped, as follows: lower limbs up to the thigh, knee, or foot only, and upper limbs up to the humerus, elbow, or hand only. Initially, the animals dropped their upper limbs up to the humerus and lower limbs up to the thigh, and were not able to raise the limbs at all (Fig. 7F). Over the course of motor recovery, they began to drop their upper limbs up to the elbow or only the hand, and lower limbs to the knee or only the foot (Fig. 7E). They also started pulling up the dropped limbs more quickly. Pulling up dropped limbs, which suggests somatosensory function recovery, was more likely to be observed in the lower than upper limbs. Somatosensory function was judged by the worst performance, even if it occurred only once.

### rhHGF promoted significant motor function recovery

**Figure 8:**
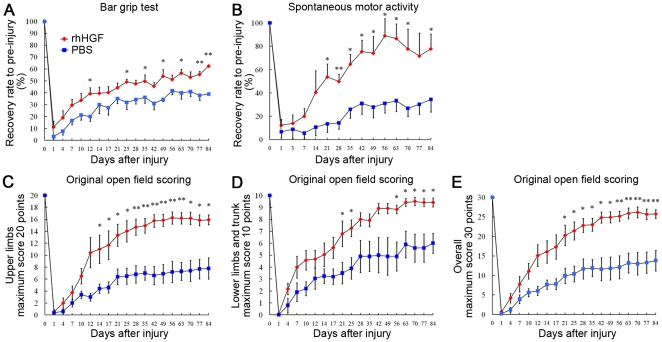
Functional recovery in rhHGF-treated marmoset SCI models. Behavioral analyses were performed by bar grip test (A), monitoring of spontaneous motor activity (B), and the original open field rating scale for the upper limbs (maximum, 20 points) (C), for the lower limbs and trunk (maximum, 10 points) (D) and combined (maximum 30 points) (E). All three methods showed significant recovery in the rhHGF group compared with the PBS group. Note that the original open field rating scale shows the smoothest recovery curve compared with the others. *P<0.05, **<0.01. (n = 6 for the rhHGF group; n = 5 for the PBS group).

The bar grip (Fig. 8A) and spontaneous motor activity (Fig. 8B) [28], [31], [33] revealed significant motor function recovery in the rhHGF group. After SCI, the bar grip strength and spontaneous movements decreased sharply, and then recovered gradually. Spontaneous motor activity tests showed greater than 100% recovery in some animals in the rhHGF group around 8 weeks after SCI. At 12 weeks after SCI, the animals had recovered 62.4±2.6% and 38.9±4.3% of their bar-grip strength, and 77.8±12.7% and 34.5±10.9% of their spontaneous motor activity, in the rhHGF and PBS groups, respectively. The evaluation of motor function by the original open field scale also showed significant differences between the two groups (Fig. 8C–E). Animals in both groups gradually recovered, reaching a plateau around 8 weeks after SCI, and the original score of the upper limbs (pre-injury score  = 20) recovered to 15.9±0.8 and 7.8±1.8 in the rhHGF and PBS groups, respectively, 12 weeks after SCI (Fig. 8C). Significant differences between the two groups were observed at most time points from 12 days after SCI in the bar grip test, from 21 days after SCI in spontaneous motor activity, and from 14 days onward after SCI in the open field upper limb scores.

### Validation of the original open field rating scale

**Figure 9:**
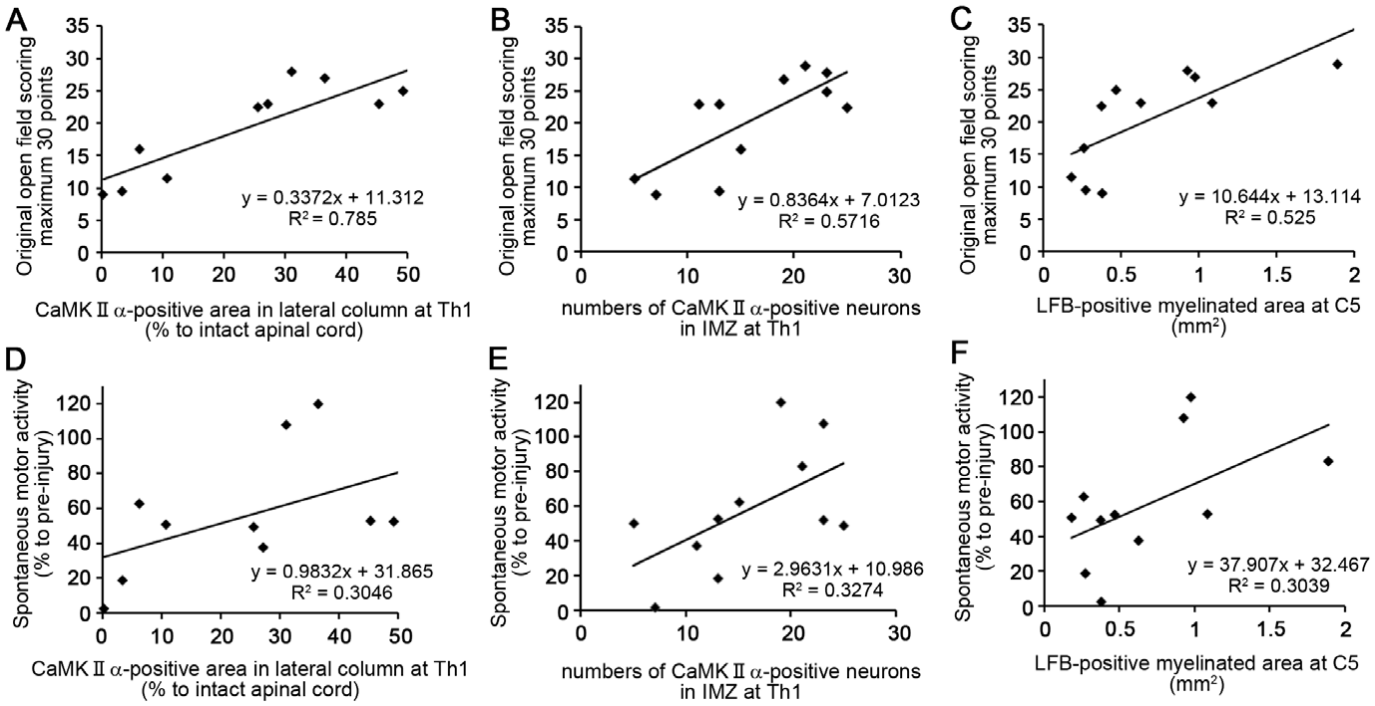
Relationship of the original open field scale and spontaneous motor activity to anatomical lesions. The original open field scale, with a maximum of 30 points, showed the greatest positive correlation with CaMK II α-positive areas in the lateral column at Th1 (A: R^2^ = 0.785) and the next most positive correlation with CaMK II α-positive IMZ neurons at T1 (B: R2 = 0.5716) compared with the four other correlations: the original open field scale and LFB-positive area at C5 (C: R^2^ = 0.525), the spontaneous motor activity and CaMK II α-positive area in the lateral column at Th1 (D: R^2^ = 0.3046), the spontaneous motor activity and CaMK II α-positive IMZ neurons at T1 (E: R2 = 0.3274) and the spontaneous motor activity and LFB-positive area at C5 (F: R^2^ = 0.3039).

Of the three methods assessing motor function (bar grip test, spontaneous motor activity, and open field scoring), our original open field rating scale yielded the smoothest recovery curve after SCI. Using this scale, the rating for the upper limbs showed a smoother recovery curve than that for lower limbs, reflecting the fact that the cervical SCI model used in this study is not a complete injury model, but rather a severe central cord syndrome model, which shows better and earlier functional recovery in the lower limbs than in the upper ones (Fig. 8C and D). To assess the validity of our open field scale, we evaluated its relationship to the anatomical lesions. Simple linear regression analyses showed that the original scale had more positive correlation with the quantitative histological analyses (Fig. 9A–C) than did the spontaneous motor activity test (Fig. 9D–F); the correlation was especially good with the CaMK II α-positive area [29] in the lateral column at Th1 (Fig. 8A; R^2^ = 0.785), suggesting that the scale closely reflected the involvement of the CST pathway.

## Discussion

We recently demonstrated that applying exogenous HGF by HSV-1 vector into the injured spinal cord of adult rats after SCI exerts significant neuroprotective and anti-apoptotic effects on neurons and oligodendrocytes to promote their survival, and enhances angiogenesis around the lesion epicenter. These multiple effects significantly reduce the area of damage and provide a better scaffold for axonal regeneration, thereby promoting functional recovery after thoracic SCI in rodents [25].

To begin to translate this HGF strategy to the treatment of human patients, here we performed intrathecal rhHGF infusion during the acute phase of cervical SCI in marmosets. Although previous clinical studies revealed that cervical SCI, which is more frequent in humans than SCI at other levels, is the main cause of manual dysfunction [34], a detailed examination of manual function following cervical SCI is difficult to obtain in rodents. A major advantage of using non-human primates in translational research is the ability to assess fine motor control recovery in the forelimbs. Another advantage of using marmosets is the similarity of their neuroanatomy to that of human beings. Some features of motor projections have undergone pronounced changes from rodents to primates.

The CST is a major pathway for voluntary movement control. It is a phylogenetically new system that appeared first in mammals and developed predominantly in primates [35], [36]. Over the course of the evolution of human and non-human primates, the CST has changed its location from the dorsal to lateral columns of the spinal cord [37] and changed its terminations. Whereas in rodents the CST axons project mainly to dorsal horn neurons and premotor spinal circuits, in primates, a significant proportion of the CST axons project to the ventral horn, and some synapse directly onto motoneurons innervating hand and forearm muscles, as direct cortico-motoneuronal (CM) connections [38].

Direct CM connections predominantly developed in higher primates such as macaque monkeys, apes, and humans [39], [40], [41]. Moreover, previous studies have suggested a strong correlation between the number of direct CM connections and the level of manual dexterity of non-human primate species [38], [39], [42], [43]; the appearance of direct CM connections is correlated with the emergence of precision grip between the thumb and the index finger, which is observed in macaque monkeys but not in marmosets. Thus, macaque monkeys have been used in many experiments because they have more advanced finger dexterity than marmosets or squirrels. In previous experiments in macaque monkeys, a unilateral transection of the spinal cord was made to interrupt the CST, including direct CM connections, at the designated cervical segment, and the recovery of precision grip was assessed after SCI [44], [45], [46], [47]. However, to evaluate and predict the therapeutic effects of interventions as human treatments, contusive injury is considered most relevant to human SCI. Furthermore, for precise assessments, the monkeys used in a translational study should be the same age, sex and body weight, which is problematic in experiments using macaque monkeys, but feasible in marmosets [48].

In this study, as shown in Figure 2, the CaMK II α-positive putative CST axons in marmosets were located in the lateral column, unlike in rodents, but projected mainly to the dorsal horn and IMZ without a visible CM connection, as in rodents, indicating the possibility that the marmoset CST is intermediate in the course of evolution from rodents to macaque monkeys and humans. Marmosets cannot perform a precise finger-thumb grip, but can be evaluated by reach-and-grasp prehensile tasks using a pen and by hand placement while walking. However, we could not evaluate these precise tasks using the previously reported bar grip test, cage-climbing test, or monitoring of spontaneous motor activity [31], [33]. Furthermore, in the spontaneous motor activity test, some animals of the rhHGF group around 8 weeks after SCI showed activity that was more than 100% of the pre-injury level, suggesting that this test does not necessarily reflect neurological recovery. Therefore, we established an original open field rating scale, which enabled us to the evaluate neurological hand performance after cervical SCI in marmosets. As shown in Figure 1, almost all of the wrist extensor and flexor motoneurons were located at a site caudal to the lesion epicenter, suggesting the usefulness of evaluating wrist and finger extension and forearm pronation, which are important for performing reach-and-grasp prehensile tasks with a pen, and for hand placement while walking.

Intrathecal rhHGF infusion significantly reduced the damaged areas and promoted functional recovery after SCI, consistent with our previous report [25]. Although HGF's anti-apoptotic, neurotrophic, and angiogenic effects were not investigated during the acute phase of SCI [25] due to the small number of animals involved in this study, the LFB-positive myelinated areas and CaMK II α-positive fibers were significantly preserved in the rhHGF group. These effects resulted in significantly better neurological hand performance, which was successfully assessed by our original open field rating scale. Notably, our original open field scale had a better positive correlation with the CaMK II α-positive area in the lateral column at the Th1 level (Fig. 9A) and with the number of CaMK II α-positive IMZ neurons at T1 (Fig. 9B) than with the LFB-positive area at the C5 level (Fig. 9C), suggesting that it primarily reflects the neurological hand performance controlled by the CST. Furthermore, compared to the spontaneous motor recovery test (Fig. 9D–F) and bar grip test, our original open field scale (Fig. 9A–C) had more a greater positive correlation with the CaMK II α-positive area, CaMK II α-positive IMZ neurons and LFB-positive areas, suggesting that this open field rating scale more precisely reflects the neurological and pathological status after cervical SCI. However, the number of surviving ChAT-positive motoneurons, which innervate forearm muscles, showed no significant differences between the HGF-treated and control groups, in contrast with our previous reports. Based on this finding, it was necessary to reexamine the direct and indirect CM pathways after cervical SCI.

Compared with the direct CM pathway, the role of indirect pathways from the cerebral cortex to motoneurons, via subcortical or spinal interneuronal systems, has received much less attention, even though such pathways contribute the majority of motoneuron input [49]. In primates, many neuroanatomical studies have shown that the majority of corticospinal fiber terminations are not distributed in lamina IX, but in the IMZ of the spinal gray matter [50], [51], where various types of interneurons are located. Intracellular recordings from motoneurons to investigate the effects of electrically stimulating the contralateral medullary pyramid (Pyr) in macaque monkeys suggest that PNs located in the C3–C4 segments can mediate the disynaptic excitation to motoneurons [52], [53]. Furthermore, an evaluation of finger dexterity (precision grip with the tips of the index finger and thumb) suggests that the C3–C4 PNs play a significant role in recovering precision grip after a CST lesion at the border between C4 and C5, in which direct CM connections to forelimb motoneurons are interrupted, while a major portion of the of the C3–C4 PN descending axons (indirect CM connections) remain intact [44]. In contrast, precision grip does not recover after a CST lesion at C1/C2, which interrupts corticospinal input to the C3–C4 PNs [54]. These findings suggest that after a CST lesion at C4/C5, indirect CM connections can be used to control the reach-and-grasp hand movements requiring a precision grip in macaque monkeys.

In the present study, we induced a C5-level contusive injury as shown in Figure 2B; that is, caudal to the C3–C4 PNs and rostral to almost all forelimb motoneurons, as shown in Figure 1. This interrupted almost all the direct CM connections to forelimb motoneurons, whereas the majority of corticospinal input to the C3–C4 PNs remained intact, as shown in Figure 2. Therefore, this cervical SCI model is appropriate for investigating the involvement and role of C3–C4 PNs in the recovery of hand dexterity. Significantly greater numbers of CaMK II α-positive interneurons in the IMZ at C4 and C6-Th1 segments were observed in the rhHGF group than the PBS group, suggesting that C3–C4 PNs played a major role in the recovery of hand movement after SCI in marmosets, as also described in macaques [44], [46], [47]. In spite of severe spinal cord damage around the lesion epicenter in the rhHGF group, motor function in the four extremities was recovered to a considerable extent, presumably by the endogenous plasticity of motor pathways, including indirect CM connections.

Beyond the CST, there are other possible mechanisms for functional recovery after SCI. First, the C3–C4 PNs could receive convergent inputs from various descending tracts, such as the rubro-, reticulo-, raphe-, and tectospinal tracts, as in rodents and cats [55], [56], [57]. Second, compared to rodents, primates engage more complex neural circuits in the parietal and frontal lobes of the cerebral cortex for even the simplest of skilled movements, suggesting that primates rely on the cortex for motor function [46], [58], [59], [60], [61], [62]; this reliance could enable greater plasticity and recovery after SCI. Third, the reticulospinal tract, located in the ventral part of the spinal cord, controls both proximal and distal arm and hand muscles [63], [64]. Experiments combining brain imaging with the pharmacological inactivation of motor cortical regions revealed time-dependent central compensatory mechanisms for finger dexterity after SCI in macaque monkeys; the recovery of finger dexterity involves the bilateral primary motor cortex during the early recovery stage, and more extensive regions of the contralesional primary motor cortex and bilateral premotor cortex during the late recovery stage [46]. In terms of clinical implications, these findings suggest that even limited sparing and regeneration of spinal projections after cervical SCI can be extremely beneficial to humans, enabling the recovery of some aspects of fine motor control.

With regard to clinical trials, methods for evaluating the spared and regenerating axons after SCI intervention are critical. If the extent and severity of the spinal cord lesion can be estimated by *in vivo* MRI techniques, including DTT [30], these analyses could provide strong predictive factors for functional recovery. In the present study, spinal tracts around the lesion site, revealed by DTT and by the intact intensity area in axial T2WI images, seemed to correlate with the spared rim of the LFB-positive myelinated area representing motor function recovery at 12 weeks after SCI; no obvious relationship between the *in vivo* images and motor function recovery was observed before 12 weeks. To improve the predictive and analytical value of these techniques, precise quantitative comparisons of *in vivo* MR images, pathological findings, and motor function over time after SCI will be necessary, especially during the acute phase of SCI (Konomi *et al.,* submitted).

We believe that intrathecal administration would also be the most effective administration route in the clinical application of rhHGF for SCI patients, because the blood-brain barrier significantly limits the translocation of rhHGF from blood to the spinal cord. We observed that the subcutaneous injection of rhHGF was ineffective in ALS rats (Funakoshi et al, unpublished result), while the intrathecal administration of rhHGF showed significant therapeutic effects in these animals [24]. In the present study, we administered 400 µg of rhHGF intrathecally over 4 weeks in marmosets, which corresponds to 0.05 mg/kg/day. Although we could not determine the HGF concentrations in the CSF of marmosets, we previously reported that the HGF levels in the spinal cord was elevated around 3-fold compared to the control level in SCI rats, when therapeutic effects of exogenous HGF were observed [25]. Therefore, we assume that the enhancement of HGF concentration in the spinal cord to no more than 3-fold above the control level would be sufficient to show therapeutic effects, and there is probably no need to increase the HGF concentration to an extremely high level. The dosage to be used in clinical applications of HGF by intrathecal administration needs to be carefully assessed. Regarding the safety of intrathecal catheter insertion, infusion from the lumbar level has a great advantage over that from the cervical or thoracic level. Moreover, catheter insertion at the lumbar level might be useful for non-operated patients with SCI. This information needs to be assessed further to establish a therapeutic dose range and the optimal administration site for use in future clinical studies in SCI patients.

Overall, intrathecal rhHGF administration after SCI significantly reduced the extent of damage to the spinal cord parenchyma, preserved the CST pathway in the lateral column, and promoted hand dexterity, presumably by involving indirect CM pathways connected by interneurons. Furthermore, marmosets did not show abnormal behaviors or signs of discomfort or pain when the examiner touched their bodies or manipulated their limbs. These findings suggest that rhHGF treatment did not induce allodynia; this was also indicated by immunohistological analysis with an anti-CGRP antibody [65]. Taken together, the present study demonstrates the validity of our original open field behavior rating scale in a contusive cervical SCI model in primates, the efficacy and safety of intrathecal rhHGF treatment for SCI in adult non-human primates and the possibility that this novel therapy may be suitable for clinical application.

## Materials and Methods

### Preparation of osmotic mini-pump containing rhHGF or PBS

Osmotic mini pumps (Alzet model 2004; nominal pumping rate 0.25 µl/hr, nominal duration 4 weeks, nominal reservoir 200 µl, Alzet, CA, USA) were filled with PBS, or with rhHGF (Kringle Pharma, Inc., Osaka, Japan) diluted with PBS so that each pump contained 400 µg of rhHGF. Each pump was connected to an intrathecal catheter (rat Intrathecal Catheter short; Alzet, CA, USA), and the apparatuses were incubated in sterile PBS at 37°C for 48 hours before use.

### Contusive SCI and intrathecal infusion of rhHGF in common marmosets

Adult female common marmosets (295–350 g; Clea Japan, Tokyo, Japan) were used (n = 6 for the rhHGF group; n = 5 for the PBS group; n = 2 for CTB injection; n = 1 for intact spinal cord). All surgeries were performed under general anesthesia induced by intramuscular injection of ketamine (50 mg/kg; Sankyo, Tokyo, Japan) and xylazine (5 mg/kg; Bayer, Leverkusen, Germany) and maintained by isoflurane (Foren; Abbott, Tokyo, Japan). The animal's pulse, arterial oxygen saturation, and rectal temperature were monitored during the surgical procedures. After a laminectomy at the C5 level, the dura mater was exposed and a 20-g weight was dropped from a height of 50 mm onto the dura using a modified-NYU impactor as reported previously [28], [31], [33]. Right after contusive SCI at the C5 level, a C7 laminectomy was carried out and an intrathecal catheter was inserted from the C7 level. An osmotic mini pump, filled with 400 µg rhHGF (HGF group) or PBS (control group) was connected to a catheter and placed in the subcutaneous space on the right side of the animal's back. The pump was left in place to deliver the total 400-µg dose of rhHGF for 4 weeks; dosage was based on previous results from intrathecal rhHGF administration in ALS rats [24], which have about the same body weight as marmosets. The animals were placed in a temperature-controlled chamber until thermoregulation was reestablished and received disinfectant treatment once a day. Manual manipulation of the fingers and joints in the four extremities was carried out twice a day and manual bladder expression was also performed twice a day until voiding reflexes were reestablished. Paralyzed animals were given adequate amounts of food and water until they recovered their ability to ingest food and water without assistance; thereafter, they had free access to food and water in the cage.

### Ethics statement

All interventions and animal care procedures were performed in accordance with the Laboratory Animal Welfare Act, the *Guide for the Care and Use of Laboratory Animals* (National Institutes of Health, USA), the *Guidelines and Policies for Animal Surgery* provided by the Animal Study Committee of the Central Institute for Experimental Animals and Keio University and the guidelines outlined by Weatherall Report,and were approved by the Animal Study Committee of Keio University (IRB approval number 09091-8).

### *In vivo* magnetic resonance imaging and diffusion tensor analysis

*In vivo* MRI and diffusion tensor studies were carried out at 1, 3, and 12 weeks after SCI in two animals from each group, four animals in total, under general anesthesia as described above. MRI was performed using a 7.0 tesla MRI, PharmaScan 70/16 (BioSpin; Bruker) with a coil dedicated for small animals; diffusion tensor analyses were performed as previously described [30]. The region of interest (ROI) was placed in the upper cervical spinal cord, and full-width DTT of the spinal cord was traced in the caudal direction.

### Retrograde cervical motoneuron labeling by **cholera toxin B subunit**

Two intact animals were anesthetized with intramuscular injections of ketamine and xylazine as described above. A small skin incision was made in the left forearm of each, and 5 µl of CTB (List Biological Laboratories, CA, USA) was manually injected into 4 points of the forearm flexor or extensor muscles. The animals were sacrificed 5 days after the CTB injection. Spinal cords were collected and immunohistological analysis performed as described below.

### Histology

Spinal cords were perfusion-fixed with 4% paraformaldehyde in 0.1 M phosphate-buffered saline (PBS), and post-fixed in the same fixative (24 hr) with 10% sucrose in 0.1 M PBS (24 hr) and 30% sucrose in 0.1 M PBS (24 hr). Spinal cord segments were embedded in an optimal cutting-temperature compound and cut into 20-µm-thick sections on a cryostat. For diaminobenzidine staining, the sections were incubated at 48°C with polyclonal anti- CaMK II α (CaMK II α; 1∶100; Zymed), polyclonal anti-CGRP (CGRP; 1∶100; Affinity), and polyclonal anti-CTB (CTB; 1∶500; Serotec) antibodies, followed by biotinylated secondary antibodies (1∶500; Jackson Immunoresearch). Biotinylated antibodies were visualized using the Vectastain Elite ABC kit (Vector Laboratories), followed by diaminobenzidine (Sigma). All images were obtained by microscopy (Axioskop 2 Plus; Zeiss, Oberkochen, Germany).

### Stereology

To quantify the CaMK II α-positive area, LFB-positive myelinated area, and CGRP-positive fiber length, axial section images were obtained and analyzed using grain counting with light intensity by a Micro Computer Imaging Device (Imaging Research Inc., St. Catharines, Ontario, Canada). Threshold values were maintained at constant levels for all analyses. After obtaining images of axial sections stained with an anti- CaMK II α antibody, the number of CaMK II α-positive neurons in the intermediate zone (IMZ) of the gray matter was counted.

### Behavioral analyses

#### Bar grip test

The bar grip test, which tests the animal's gripping reflex (the motion undertaken when attempting to grasp an object placed in front of their eyes) and evaluates the grip power, was carried out as previously reported [28], [31], [33]. The same device (220-mm wide, 500-mm deep, and 400-mm high, with a bar diameter of 2.5 mm in a 1×3 [70 mm×100 mm] grid pattern) was used in the present study. The test was carried out three times a day. The percentage of the maximal grip strength relative to the mean strength before the injury was calculated on each day after the injury.

#### Spontaneous motor activity test

Spontaneous motor activity tests were carried out as previously reported [28], [31], [33]. We used cages (350-mm wide, 500-mm deep, and 500-mm high) equipped with infrared sensors (Murata Manufacturing Corp., Nagaokakyo, Kyoto, Japan) on the ceiling to continually record the marmosets' 3D motion. Our system utilized a passive thermographic infrared sensor to monitor heat emitted from the animals. The 3D localization of the heat source was monitored, and a change in this localization was recorded as movement. Each animal's data were recorded and monitored hourly on a computer, and the activity measured after the SCI was calculated as a percentage relative to that before the injury.

#### Original open field rating scale

To develop an original open field rating scale, the natural course of motor function recovery after cervical SCI was precisely observed in 6 marmosets before starting experiments using rhHGF. Two blinded examiners participated in all the open field tests and were positioned across from each other to observe both sides of the marmoset. Marmosets were tested alone for 5 minutes on the floor and in the cage 3 times per week until 4 weeks after SCI, and once per week thereafter. During the open field test, marmosets were encouraged by slight tapping when they remained stationary for longer than 15 seconds. In cases of borderline locomotor performance or disagreement between examiners, scores indicating the greater deficit were assigned. All lower limb movements were counted except those that were obviously part of a reflex (i.e., spastic extensive bilateral flexion, or extension of the hip, knee, or ankle joints). The bilateral upper limbs were assessed separately, and the score was defined as the mean of the bilateral scores for motor function.

### Statistical analyses

All data are reported as the mean±SEM. In all the histological examinations, an unpaired two-tailed Student's t-test was used for single comparisons between the rhHGF and PBS groups. The results of the original open field rating scale, bar grip test, and spontaneous motor recovery test were analyzed using the Mann-Whitney U-test. To validate the original open field rating scale, correlations between the results of this scale and the spontaneous motor activity or histological findings (CaMK II α-positive areas in the lateral column at Th1, CaMK II α-positive neurons in IMZ andLFB-positive myelinated areas at C5) were analyzed by simple linear regression analyses.
